# Alleviation of Hepatic Steatosis by Alpha-Defensin Is Associated with Enhanced Lipolysis

**DOI:** 10.3390/medicina59050983

**Published:** 2023-05-19

**Authors:** Emad Maraga, Rifaat Safadi, Johnny Amer, Abd Al-roof Higazi, Rami Abu Fanne

**Affiliations:** 1 Department of Clinical Biochemistry, Hadassah Hebrew University Hospital, Jerusalem IL-91120, Israel; 2 Liver Unit, Hadassah Hebrew University Hospital, Jerusalem IL-91120, Israel; 3 Department of Cardiology, Hillel Yaffe Medical Center, Rappaport Faculty of Medicine, Technion-Israel Institute of Technology, Haifa 3200003, Israel

**Keywords:** non-alcoholic fatty liver disease (NAFLD), alpha-defensin, human neutrophil-derived alpha-defensin (HNPs), fibrosis, steatosis

## Abstract

*Background and Objectives*: The neutrophilic peptide, alpha-defensin, is considered an evolving risk factor intimately linked with lipid mobilization. It was previously linked to augmented liver fibrosis. Here, we assess a potential association between alpha-defensin and fatty liver. Materials and *Methods*: A cohort of transgenic C57BL/6JDef^+/+^ male mice that overexpress the human neutrophil-derived alpha-defensin in their polymorphonuclear neutrophils (PMNs) were assessed for liver steatosis and fibrosis development. Wild type (C57BL/6JDef.Wt) and transgenic (C57BL/6JDef^+/+^) mice were maintained on a standard rodent chow diet for 8.5 months. At the termination of the experiment, systemic metabolic indices and hepatic immunological cell profiling were assessed. *Results*: The Def^+/+^ transgenic mice exhibited lower body and liver weights, lower serum fasting glucose and cholesterol, and significantly lower liver fat content. These results were associated with impaired liver lymphocytes count and function (lower CD8, NK cells, and killing marker CD107a). The metabolic cage demonstrated dominant fat utilization with a comparable food intake in the Def^+/+^ mice. *Conclusions*: Chronic physiological expression of alpha-defensin induces favorable blood metabolic profile, increased systemic lipolysis, and decreased hepatic fat accumulation. Further studies are needed to characterize the defensin net liver effect.

## Core Tip:

NAFLD is a multifactorial disease; however, fat deposition is largely considered a prerequisite for the development of NAFLD/NASH. The role of inflammation in the induction of NAFLD is increasingly appreciated. Alpha-defensin is the major neutrophilic peptide that is directly involved in cholesterol shuttling and deposition in tissues and has recently bseen linked to liver fibrosis. In the current study, we prove a negative association between alpha-defensin expression and liver steatosis. We show this finding to be at least partially mediated through dominant fat metabolism in defensin mice. Lastly, we provide several clues demonstrating that the defensin net effect is a curse rather than a blessing.

## 1. Introduction

Non-alcoholic fatty liver disease (NAFLD) is the most common form of liver disease worldwide. NAFLD is a complex disease associated with hepatic steatosis, which is affected by a variety of environmental and genetic factors [1,2,3,4,5]. It is largely considered a hepatic manifestation of the metabolic syndrome. This disorder encompasses a wide range of diseases, from simple steatosis, which is relatively benign, to hepatic inflammation, hepatocyte injury, and fibrosis, a syndrome referred to as non-alcoholic steatohepatitis (NASH), that can progress to cirrhosis [6,7,8,9].

Currently, there are several non-invasive diagnostic tools used for the assessment of NAFLD including biochemical markers; alanine aminotransferase (ALT) and aspartate aminotransferase (AST) as non-invasive indicators of NAFLD; as well as histology and imaging modalities (CT, MRI, US). Previous studies have shown a high prevalence of NAFLD in individuals with hepatomegaly or type 2 diabetes (T2D), both associated with elevated levels of blood glucose and insulin resistance [10]. Nevertheless, the pathogenesis of NAFLD is partially elucidated.

Despite the definite role of over-nutritious and sedentary lifestyles [11] in NAFLD induction, most obese people are NAFLD-free, emphasizing the importance of individual susceptibility. However, it is puzzling why some individuals with NAFLD develop advanced histological features and progress to cirrhosis, whereas others with comparable risk profile have simple steatosis with minimal or no disease progression.

Previous reports discussed the impact of chronic inflammation in the initiation, maintenance, and progression of NAFLD [12,13]. In this interplay, the net role of neutrophils is still controversial; some suggested that insulin resistance may promote the infiltration of neutrophils into the liver through IL-8 and growth-related oncogene alpha, resulting in NAFLD/NASH development [14]. Myeloperoxidase, a major enzyme from the azurophilic granules of neutrophils, catalyzes the formation of oxidized phosphatidylcholine and enhances the progression of hepatic steatosis in patients with NASH [15]. In addition, a deficiency of myeloperoxidase diminishes fat accumulation and fibrotic change in the livers of low-density lipoprotein-deficient mice that are fed a high-fat diet [16].

Alpha-defensin is the major neutrophilic peptide with non-oxidant induced microbicidal activity against multiple pathogens and chronic diseases [17,18]. Recently, hepatic fibrosis was aggravated in transgenic pan-tissue, an alpha-defensin expression model, through inducing hepatic stellate cell proliferation [19]. Interestingly, the authors reported a null effect on hepatic steatosis that was ascribed to advanced fibrosis.

Alpha-defensins comprise 40–50% of the content of neutrophil azurophilic granules [20,21]. We have already studied the role of alpha-defensins in low-density lipoprotein (LDL) shuttling, and atherogenesis using transgenic mice expressing human alpha-defensins in their polymorphonuclear leukocytes (Def^+/+^). The Def^+/+^ mice developed dimers (alpha-defensin·LDL) that accelerated the clearance of LDL from the circulation; augmented hepatic, vascular deposition and retention of LDL; induced endothelial cathepsins; and increased the development of lipid streaks in the aortic roots while being fed a regular diet and had normal plasma levels of LDL [22].

The above effect of alpha-defensin on cholesterol shuttling to the liver prompted us to investigate the liver phenotype of long-term, non-provoked, physiological alpha-defensin expression. The results show that the Def^+/+^ mice exhibited lower body and liver weights, lower serum glucose and cholesterol levels, higher serum alanine aminotransferase and triglycerides, and a decreased steatosis. These findings suggest that the chronic physiological expression of alpha-defensin is associated with anti-steatotic liver changes.

## 2. Materials and Methods

### 2.1. Mice

Transgenic C57BL/6J^Def+/+^ male mice overexpressing human neutrophil-derived alpha-defensin in their polymorphonuclear neutrophils (PMNs) were previously characterized [21]. Briefly, the (C57BL/6-Def^+/+^) transgenic mice expressing alpha-defensin-1 in their neutrophils were generated using the bacterial artificial chromosome as previously described [23]. The construct carries four full-length copies of the alpha-defensin-1 gene (DEFA1), and one truncated copy of the alpha-defensin-3 gene (DEFA3).

The transgenic mice and their control littermates (C57BL/6^Def.Wt^) were obtained from University of Pennsylvania, Philadelphia, USA, and housed in the small animal facility at the Hebrew University of Jerusalem under specific pathogen-free (SPF) conditions. Animal care and experimentation was conducted in accordance with protocols approved by the Institutional Animal Care and Use Committee (IACUC) of the Hebrew University, which adheres to Israeli guidelines and follows the NIH/USA animal care and use protocols. Mice were housed on hardwood chip bedding in individual ventilation cages (IVC) under a 12 h light/dark cycle at 21–23 °C, given tap water and standard rodent chow diet that contained 4.5% fat (PMI5010, Harlan, Rehovot, Israel) ad libitum since weaning day (3 weeks old) until the end of the experiment at the age of 8.5 months. In each experimental group, 6–8 mice of the C57BL/6 transgenic (Def^+/+^) and its control (WT) were assessed. This study was conducted in the years 2017–2018. Notably, the expression of alpha-defensin in the transgenic mice was confirmed by measuring the concentrations of alpha-defensin in plasmas and sera via enzyme-linked immunosorbent assay (ELISA) as described previously (22).

### 2.2. Plasma Analysis

Blood samples were collected at the age of 8.5 months, after 12 h fasting, via direct cardiac puncture. Plasma glucose, triglycerides, total cholesterol, alanine aminotransferase (ALT), and aspartate aminotransferase (AST) levels were measured using the Cobas^®^ 6000 analyzer series (ROCHE diagnostics). Plasma samples were diluted 1:10 with PBS for measurement of ALT and AST.

### 2.3. Quantification of Total Hepatic Lipid

Total hepatic lipid was determined using a protocol adapted from Mopuri et al. [24] with minor modifications. Livers were obtained at the ages of 4 months and 8.5 months and were kept at −80 °C. Frozen liver tissue (100 mg) was homogenized in 4 mL of 2:1 chloroform-methanol solution, at 10,000–15,000 rpm, under 4 °C cooling for 10–30 s, until a homogenous texture was obtained. Samples were kept for 2 h at 4 °C, and then centrifuged at 5000 to 7000 rpm for 10 min to facilitate the separation of the upper phase (aqueous methanol dragging) and the lower phase (chloroform phase) containing the lipids. A volume of 1 mL of the chloroform phase was transferred into a tube previously weighed, and the solution was evaporated by drying using a nitrogen stream. The tube was weighed again, and the amount of fat was extracted. Total lipid per 100 mg liver tissue was calculated. Finally, lipids were dissolved in isopropanol and triglycerides measured using spectrophotometric method.

### 2.4. Liver Fat Fraction Quantification Using EchoMRI

The total ex vivo liver fat contents were evaluated using the EchoMRI™-100H (Echo. Medical Systems, Houston, TX, USA) at 4 and 8.5 months. The mice were sacrificed via cervical dislocation and the livers were completely removed for EchoMRI evaluation. The tube was thoroughly sanitized using 10% bleach solution and then rinsed and dried after each use.

### 2.5. Tissue Processing and Histological Analyses

All animals were housed in a temperature-controlled room (23–24 °C) on a 12 h light/dark cycle and had free access to food and water. Mice were sacrificed via cervical dislocation. After fixation in 10% neutral buffered formalin, a slice of the median lobe of the liver was trimmed, processed, sectioned into slices approximately 5 µm thick, and mounted on a glass slide. Hematoxylin and eosin stains, liver immunofluorescence staining with Hsp60 and β-Catenin, and electron microscope images were used for morphological analyses, and Masson’s trichrome and Sirius red stains (0.1% Sirius red F3B in saturated picric acid (both from Sigma, Inc. (St. Louis, MO, USA))) were used for assessment of hepatic fibrosis. Histopathological analysis was performed by a pathologist who was not exposed to the study groups. Data were derived from blinded analysis of 5 sections from each of the 8 animals in each group. We used the NAFLD activity score (NAS) system to estimate steatosis grade and fibrosis stage.

### 2.6. Cell Isolation, Staining, and Flow Cytometric Analysis

The spleen was homogenized, and lymphocytes were washed and counted before staining for flow-cytometric analysis. Intrahepatic lymphocytes were isolated via perfusion of the liver with digestion buffer. After perfusion, the liver was homogenized and incubated at 37 °C for 30 min. The digested liver cell suspension was centrifuged to remove hepatocytes and cell clumps. The supernatant was then centrifuged to obtain a pellet of cells depleted of hepatocytes to a final volume of 1 mL. Lymphocytes were then isolated from this cell suspension using 24% metrizamide gradient separation. Liver natural killer (NK) cells, CD4+, and CD8+ subsets were isolated using DYNAL Biotech kits according to manufacturer’s instructions.

### 2.7. Metabolic Caging Analysis

Metabolic rate of the control and Def^+/+^ mice on standard diet (STD) was measured using the Promethion Metabolic Phenotyping System by Sable Systems International, by incorporating sub-systems for open-circuit indirect calorimetry, feeding, water intake, activity, running wheel, body mass, and core temperature measurements in conventional live-in home cages, mice experienced minimized stress (Sable Instruments, Inc., Las Vegas, NV, USA); this fully automated system is the “one-test” solution for simultaneous multi-parameter assessment for any metabolic, behavioral, and physiological research. While in the metabolic chambers, the mice had free access to food and water. We analyzed 8 individually caged mice at the same time. Mice were housed in these cages for 7 days. These were conventional live-in home cages with regular bedding. The parameters provided by the metabolic cages included food intake, respiratory quotient, total energy expenditure, fat oxidation, and carbohydrate oxidation.

### 2.8. Statistical Analysis

Continuous variables are presented as a mean and standard deviation, and categorical data are presented as percentages. Categorical variables were compared using Pearson’s chi-square test, while continuous variables were compared using Student’s *t*-test. All statistical analyses were performed on IBM SPSS version 26. Statistical significance was set at the 2-tailed 0.05 level, without multiplicity adjustment.

## 3. Results

### 3.1. Plasma Biochemical Markers

**Figure 1:**
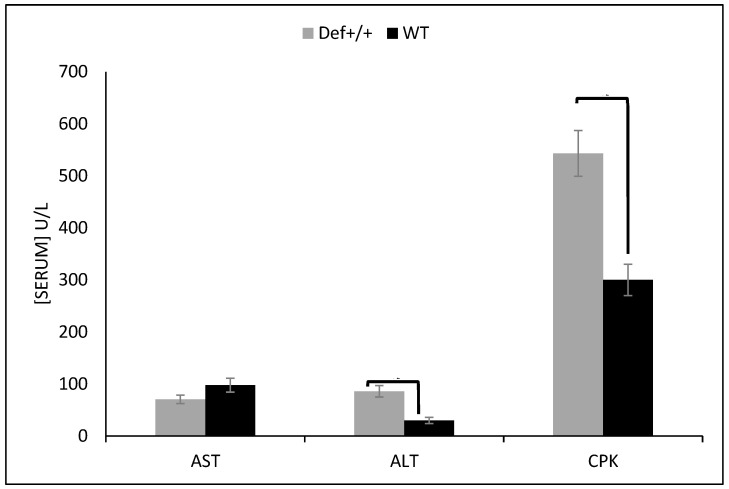
Fasting serum values of three different liver enzymes, including aspartate transaminase (AST), alanine aminotransferase (ALT), and creatine phosphokinase (CPK) in wild type (WT) and Def^+/+^ transgenic mice. Eight mice aged 8.5 months were assessed in each experimental group. Values of WT and Def^+/+^ transgenic mice are presented in the black and gray bars, respectively. *x*-axis shows the three enzymes, and the *y*-axis shows the mean values in U/L with standard deviation presented on the bars.

After 12 h of fasting, the plasma levels of the total cholesterol and glucose were significantly lower in the 8.5-month-old Def^+/+^ mice than the WT mice that were fed a standard chow diet (137 ± 8 vs. 247 ± 13 mg/dL, *p* < 0.05 and 72.5 ± 3.5 vs. 94.8 ± 5 mg/dL, *p* < 0.05, respectively). Moreover, the Def^+/+^ mice showed lower body (21.9 ± 0.55 gr vs. 31.4 ± 0.96 gr, *p* < 0.05) and liver (995 ± 75 mg vs. 1389 ±106 mg, *p* < 0.05) weights, lower levels of aspartate aminotransferase (50.6 ± 5.4 vs. 97.7 ± 5.3, *p* < 0.05), and higher levels of CPK (543 ± 44 vs. 300 ± 30, *p* < 0.05), alanine aminotransferase (86 ± 11 vs. 30 ± 6, *p* < 0.05) (Figure 1), and triglycerides (59 ± 9 vs. 45 ± 5.5 mg/dL, *p* < 0.05). The liver synthetic function (albumin and prothrombin) was equally preserved in the Def^+/+^ mice, suggesting a preserved nutritional status of both groups.

### 3.2. Quantification of Total Hepatic Lipid

**Figure 2:**
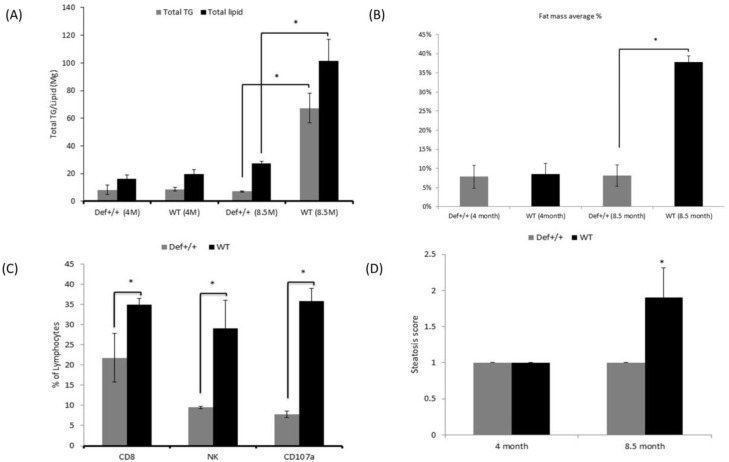
(**A**) Average ratio of total lipid content and total triglycerides (TG) per liver in Def^+/+^ transgenic and WT mice at the ages of 4 and 8.5 months. Eight mice in each experimental group were assessed. Values of WT total lipid content and total TG are presented in the black and gray bars, respectively. (**B**) Percentage (%) of liver fat mass average based on EchoMRI analysis in the WT and Def^+/+^ transgenic mice at ages of 4 and 8.5 months. Eight mice were assessed in each experimental group. *x*-axis shows the experimental groups, and the *y*-axis shows the percentage (%) of liver fat mass with standard deviation presented on the bars. (**C**) Percentage presentation of the total lymphocytes of pro-fibrotic markers in the serum as an indicator of fibrosis in the liver in the WT and Def^+/+^ transgenic mice at 8.5 months of the experimental period. The three markers are CD8 cells, the anti-fibrotic NK lymphocytes, and the NK cell activation marker CD107a. Six mice in each experimental group were assessed. Values of WT and Def^+/+^ transgenic mice are presented in the black and gray bars, respectively. (**D**) Main steatosis grade in the WT and Def^+/+^ transgenic mice at the ages of 4 and 8.5 months. Six mice in each experimental group were assessed. Values of WT and Def^+/+^ transgenic mice are presented in the black and gray bars, respectively. *x*-axis shows the WT and Def^+/+^ transgenic mice and the *y*-axis shows the steatosis grade with standard deviation presented on the bars. Asterisk (*) indicates *p* < 0.05 between WT and Def^+/+^ transgenic mice.

The hepatic profiles of the fat accumulation of the mice livers are given in Figure 2A. At 4 months of age, the hepatic lipid composition was similar in both groups. At 8.5 months of age, the lipid content in Def^+/+^ mice was marginally affected with even slight, non-significant decrease in the total TG content. However, the total lipid content and total triglyceride/liver were significantly higher in the WT group (*p* < 0.05) at the age of 8.5 months.

### 3.3. Liver Fat Fraction Quantification using EchoMRI

The EchoMRI results of the fat mass composition in the livers were in line with the biochemical findings. The MRI detected slightly higher fat mass percentage in the 4-month-old WT mice, which significantly increased at the age of 8.5 months, reaching 23.3% ± 3 compared to the 10.7% ± 1 (*p* < 0.05) fat mass in the Def^+/+^ mice (Figure 2B).

### 3.4. Lymphocyte Isolation

The immune system was shown to play a key role in the development of liver fibrosis. Different subtypes of lymphocytes are involved in liver fibrosis as well as in the clearance of necrotic cells during inflammation.

In the present study, the percentage of the pro-fibrotic CD8 cells (21.75 ± 6% vs. 35 ± 1.4%, *p* < 0.05), the anti-fibrotic NK lymphocytes (9.5 ± 0.3% vs. 29 ± 7.1%, *p* < 0.05), and the NK cell activation marker CD107a (7.8 ± 0.9% vs. 35.8 ± 3.2%, *p* < 0.05) were significantly lower in the Def^+/+^ mice aged 8.5 months (Figure 2C).

### 3.5. Tissue Processing and Histological Profile

**Figure 3:**
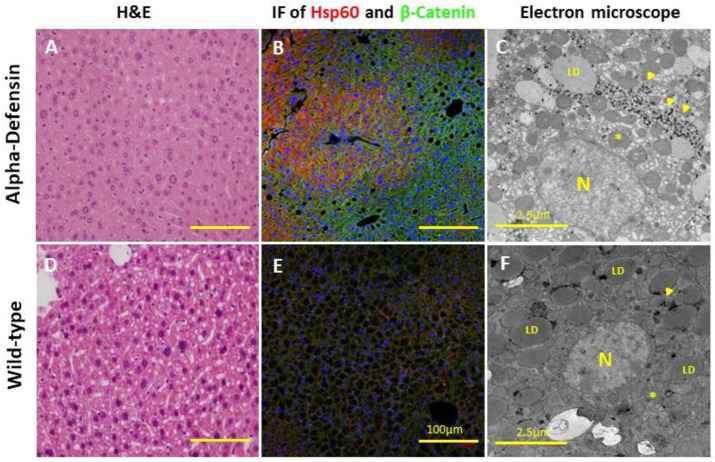
(**A**–**C**) H&E, immunofluorescence (×400, Scale bar: 100 µm), and electron microscopy images, respectively, of Def^+/+^ liver slides at the age of 8.5 months (6 mice per group). (**D**–**F**) H&E, immunofluorescence, and electron microscopy images, respectively, of WT liver slides at the age of 8.5 months (6 mice per group). *, mitochondria; N, nucleus; LD, lipid droplet; arrow, glycogen.

**Figure 4:**
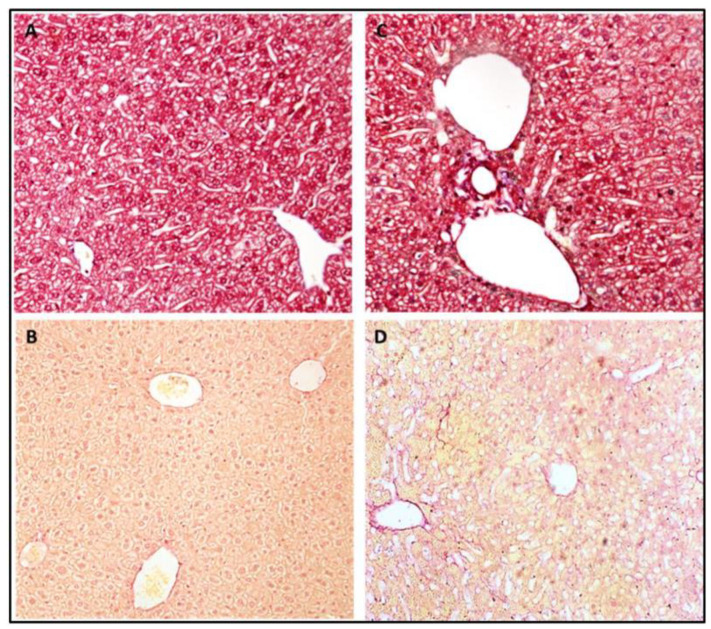
Masson trichrome blue and Sirius red liver staining of 8.5-month-old WT mice (**A**,**B**) and their Def^+/+^ transgenic age-matched group (**C**,**D**) of mice.

The histological examination demonstrated a low steatosis grade in the Def^+/+^ transgenic mice relative to the age-matched WT mice (1 ± 0.0 vs. 1.9 ± 0.4, *p* < 0.05, respectively), with the latter exhibiting a diffuse mixed macro and microvesicular steatosis (Figure 3). Notably, both mice groups started with minimal steatosis at the early age of 4 months (Figure 2D). The Sirius red and Masson blue staining for fibrosis disclosed mildly increased fibrosis stain in the Def^+/+^ group, with a near absence of fibrosis in the WT group (Figure 4). In addition, the electron microscopy demonstrated a lower fat accumulation, and a higher density of glycogen deposition in the Def^+/+^ mice.

### 3.6. Metabolic Caging Analysis

**Figure 5:**
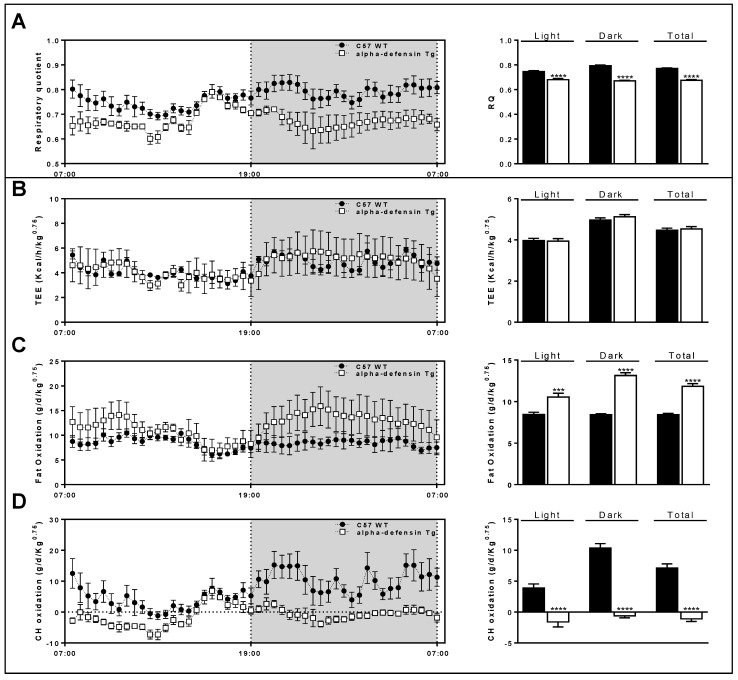
Def^+/+^ mice show different respiratory profile compared to their WT littermates. Alpha-defensin transgenic mice demonstrate lower respiratory quotient (**A**); overexpression of alpha-defensin does not change total energy expenditure (TEE; **B**), but significantly increases fat oxidation (**C**) and lowers carbohydrate oxidation (**D**). Method: Mice were monitored using the Promethion High-Definition Behavioral Phenotyping System (Sable Instruments, Inc., Las Vegas, NV, USA) over a 24 h period. Effective mass was calculated by power of 0.75. Data are mean ± SD from 4 mice per group. *** *p* < 0.001 vs. C57 WT, **** *p* < 0.0001 vs. C57 WT.

The Def^+/+^ mice show a different respiratory profile compared to their WT littermates. The alpha-defensin transgenic mice demonstrated a lower respiratory quotient (Figure 5A). Moreover, the overexpression of alpha-defensin does not change the total energy expenditure (TEE; Figure 5B). On the other hand, the overexpression of alpha-defensin is reflected by a higher fat oxidation (Figure 5C) and a lower carbohydrate oxidation (Figure 5D).

## 4. Discussion

Non-alcoholic fatty liver disease (NAFLD) is a worldwide looming epidemic. It is associated with a wide range of pathological manifestations at the liver level [25], as well as extrahepatic complications, including cardiovascular disease [26].

Effective therapeutic approaches for NAFLD patients are limited, and lifestyle modification remains the mainstay of treatment. Hence, the identification of innovative targets and therapies is imperative.

Over the last years, the appreciation of the role of inflammation in NAFLD has burgeoned. Compelling evidence indicates that insights gained from the link between inflammation and NAFLD can yield predictive and prognostic information of considerable clinical utility. Previously, we described the role of alpha-defensin dimerization with LDL in accelerating cholesterol clearance from the circulation, and promoting its internalization, deposition, and retention in the vessel wall, as well as in the liver [22]. Alpha-defensin overexpression eventually induced atherosclerosis despite normal levels of plasma cholesterol, and lower glucose levels. In vitro, alpha-defensins were implicated in the activation of macrophages [27,28], and in the production of TNFα. The latter was proven to play a critical role in the induction and progression of NAFLD [29,30,31].

The above findings prompted our initial speculation for the Def^+/+^ mice as a potential trackable link between inflammation and the development of steatosis. Animal models used to study NAFLD mostly involve diets and different insults that do not simulate the metabolic or inflammatory context of human NAFLD. In this paper, we test the unprovoked, long-term effect of alpha-defensin expression on liver fat deposition. The old WT mice exhibited moderate liver steatosis, as opposed to negligible fat accumulation in the Def^+/+^ mice; this finding was concordant with significantly lower Def^+/+^ body weight.

In an attempt to reconcile the reduced liver fat content, we used the metabolic cages for metabolic and behavioral phenotyping. We found a similar food intake of the WT and Def^+/+^ mice. Surprisingly, although alpha-defensin overexpression did not affect the total energy expenditure, it prompted a significantly higher fat oxidation and lower carbohydrate consumption. The reduced carbohydrate utilization might explain the abundance of glycogen in the Def^+/+^ mice livers seen in EM.

The Ibusuki group [19] used a pan-tissue alpha-defensin expression model plus a CDAA diet and proved a significant fibrotic effect of alpha-defensin with a null effect on steatosis at the age of 17 weeks. The current study mainly demonstrates a substantial anti-steatotic effect of physiological alpha-defensin expression, which is obviously not a result of advanced liver fibrosis. We believe both “hits” of non-physiological alpha-defensin overexpression and a CDAA pro-fibrotic diet amplified the fibrotic effect of alpha-defensin in the Ibusuki study. In the same line, Ibusuki and colleagues measured lower levels of serum cholesterol and triglycerides in the alpha-transgenic mice that were ascribed to advanced hepatic fibrosis. In our model, we found using the blood coagulation tests that more sensitive clues of hepatic dysfunction/fibrosis were preserved in the Def^+/+^ mice. The described low cholesterol is potentially the result of active cholesterol shuttling and augmented utilization.

Golabi and colleagues [32] recently introduced the entity of “lean NAFLD”; this group comprises 10–20% of NAFLD patients, also termed metabolically unhealthy non-obese NAFLD. Our model of the physiological expression of alpha-defensin, free from dietary insults, ends with lean, normoglycemic and normolipidemic non-fatty liver mice. These lower indexes are neither related to energy expenditure nor to decreased food intake. Innovatively, the Def^+/+^ mice are dominant fat metabolizers with extremely low glucose consumption. This finding may explain both the lower fat contents with higher “lipolysis derived” plasma TG levels and residual glycogen deposits in the Def^+/+^ mice livers via a mechanism that is yet to be clarified.

## 5. Conclusions

To our knowledge, this is the first study to show that alpha-defensin might evolve as a hepatic anti-steatotic factor. As fat accumulation in the liver is considered a prerequisite for the development of NAFLD/NASH, future research investigating the metabolic impacts of alpha-defensin seems imperative.

## Data Availability

All data generated or analyzed during this study are included in this published article.
